# DEFINING AND CHARACTERIZING MAINTENANCE: RESULTS FROM A LITERATURE REVIEW

**DOI:** 10.1093/geroni/igad104.1289

**Published:** 2023-12-21

**Authors:** Rahma Ajja, Anna Thorpe, Bridget Smeekens, Jaime Hughes

**Affiliations:** Wake Forest University School of Medicine – Winston-Salem, North Carolina, Winston Salem, North Carolina, United States; Wake Forest University School of Medicine, Winston-Salem, North Carolina, United States; Wake Forest University School of Medicine, Winston-Salem, North Carolina, United States; Wake Forest School of Medicine, Winston-Salem, North Carolina, United States

## Abstract

Leading models of health behavior change define maintenance as sustainment of the target behavior over time. Although behavior change interventions for physical activity have shown to be effective in older adults, maintenance of activity levels after an active intervention period is rarely examined. This literature review aimed to identify the following: (1) frequency with which maintenance is described and measured in the scientific literature; (2) intervention techniques utilized to promote maintenance; and (3) common barriers and facilitators to maintenance. Systematic searches of electronic databases (Embase, CINAHL, PubMed) identified 20 eligible studies (conducted in United States, single component physical activity intervention with 1+ follow-up period(s) beyond post-treatment assessment). Six studies reported maintenance in the title and only 8 included a theoretical model or framework [Social Cognitive Theory (N=5), Theoretical Domains Framework (N=1), Attribution Theory (N=1), Three-Level Hierarchical Framework (N=1)]. No studies defined maintenance. The majority of studies (N=16) provided only 1 follow-up after the post-treatment assessment (range: 4 weeks – 18 months). One-half of studies described specific intervention strategies to support maintenance, including goal-setting, personalized feedback, and social support. Six studies described barriers, including costs, aversion to activity or environment, low motivation, time constraints, and self-efficacy. Only two studies reported facilitators to maintenance, including knowledge, confidence, self-efficacy, and social support. These findings highlight the gap in health behavior scientific literature addressing maintenance of physical activity behaviors following structured interventions for older adults.

